# Annual Session of the Association of Medical Superintendents of American Institutions for the Insane

**Published:** 1868-06

**Authors:** 


### Annual Session of the Association of Medical Superintendents of American Institutions for the Insane.

The Medical Superintendents of the American Institutions for the Insane commenced their twenty-second annual session at the American House in Boston, June 3d, at 10 o'clock.

The first subject taken up by the Association was "The project for a general law for determining the legal relations of the Insane," and Dr. Ray was called upon to make some remarks in regard to the history and treatment of this question. Dr. Ray alluded to the necessity for having a general law which should be in force in every State. In order to facilitate the work in preparing such a law. a committee was some time ago appointed and the laws in the different States were considered and compared. This committee reported at the meeting at Washington in 1864. The points covered in the law were partially discussed at that meeting, and since that time the whole matter has been in abeyance. Experience is every year demonstrating more aud more the necessity of such a law, and cases are constantly occurring which urge the subject upon the attention of all persons interested in the treatment and cure of the insane. The general sentiment of the community is that no one class of persons ought to have entire and absolute control in regard to cases of insanity. Such a law as is proposed need not cover all the points in question in regard to the treatment of insanity, and the principal thing to be considered is in regard to the committal of persons to the insane asylums. He would not advocate the relinquishment of the authority which has long been vested by common consent in the families and friends of insane persons, but some legal process is necessary for the care of those persons who have no families, or whose familres do not care to take such a step. For these and kindred cases it is very desirable that the matter should be attended to legally and properly. Such a law, too, is necessary for the pauper insane, for the authorities who have them in charge are generally likely to avoid the expense of committing them to asylums as long as possible. Such a law, too, is necessary for the care of the vagrant insane, who have no regular residence, and whose confinement is an act of humanity to themselves and a means of safety for the community.

At the re-assembling of the Association in the afternoon, Prof. Charles A. Lee of New York, made an address of welcome as a delegate from the American Medical Association. He alluded in the strongest terms to the progress that has been made in recent years in the treatment of the insane, and congratulated the Association upon all that its members had been able to do for the advancement of one of the most important departments of medical science. The address was very cordial in its tone, and the Association manifested great pleasure at the interest taken in it and its work by the Association represented by Dr. Lee.

Evening Session.—The consideration of the question of making provision for commitments of patients to Insane Asylums was resumed, when the Association re-assembled at 8 o'clock. Dr. Earle withdrew the motion for the postponement of the discussion until next year, and Drs. Chipley and Hills withdrew the substitutes for the first section of the proposed object of a general law, which had been offered by them. Dr. Hills then offered the following, which was adopted as a sense of the Association by a yea and nay vote, Dr. Harlow of Maine alone voting in the negative:

Insane persons may be placed in a hospital for the insane by their legal guardians, or by their relatives and friends, in case they have no guardians, but never without the certificate of one or more responsible physicians. after a personal examination made within one week of the date thereof; aad this certificate to be duly acknowledged before some magistrate or judicial officer, who shall certify to the genuineness of the signature and of the respectability of the signer.

The remaining sections of the proposed model for a law, as reported by Dr. Ray, were then successively considered and adopted, most of them with but little change of form and with little debate. The first section was the only one upon which there was any very decided diversity of opinion, and the substitute for that section finally adopted seemed to give general and almost unexpected satisfaction to all the members of the Association.

